# Museum-Based Education in Health Professions Learning: A 5-Year Retrospective

**DOI:** 10.5334/pme.1448

**Published:** 2024-11-25

**Authors:** Kain Kim, Sujal Manohar, Meher Kalkat, Kayla Iuliano, Margaret S. Chisolm

**Affiliations:** 1 Emory University School of Medicine, Atlanta, Georgia, US; 2 Baylor College of Medicine, Houston, Texas, US; 3 Johns Hopkins School of Medicine, Baltimore, Maryland, US; 4 Epidemiology Department at the Geisel School of Medicine at Dartmouth, Hanover, New Hampshire, US; 5 Psychiatry and Behavioral Sciences, and of Medicine, at Johns Hopkins University School of Medicine, Baltimore, Maryland, US

## Abstract

A growing evidence base suggests that the integration of museum-based activities into health professions education can contribute to learner resilience and wellbeing, promote capacity for patient-centered care, and encourage equity in learning environments. However, the styles and methods for implementing museum-based programs vary widely across different institutions. This retrospective leverages the lessons learned from 5 years of experience implementing museum-based programs at one large academic institution to examine the various operational and logistical considerations in implementing a museum-based program for health professions learners. These efforts are intended to support the Association of American Medical Colleges and National Academies of Sciences, Engineering, and Medicine’s aims to formally integrate the arts and humanities into health professions education. We structure our recommendations under the subheadings of program form, audience, function, and evaluation, with hopes of providing medical educators with starting guidelines that are broadly adoptable across different institutions.

## Introduction

In the past five years, the World Health Organization, US National Academies of Sciences, Engineering, and Medicine, and the Association of American Medical Colleges (AAMC) have issued urgent calls for the integration of the arts and humanities into medical education [1,2,3]. The AAMC’s report on the Fundamental Role of the Arts and Humanities in Medical Education (FRAHME), informed by a scoping review, suggests that the arts and humanities provide a novel way to develop clinically relevant skills and attitudes, such as empathy and tolerance for ambiguity [3,4].

Within this realm of arts and humanities curricula, museum-based education has been shown to contribute significantly to health professions learning across all four domains of the Prism Model: skill mastery, perspective-taking, personal insight, and social advocacy [5]. Museum-based education encompasses a broad range of arts activities that can be tailored to desired educational outcomes. Since 2019, Johns Hopkins University (JHU) has offered many museum-based programs for health professionals, both in-person and online [5,6,7,8,9]. These have been designed for various learner levels – including pre-health undergraduates, preclinical medical students, and clinical learners – to foster the development of clinically relevant skills and attitudes (Table 1) [6] and professional identity formation [5,9].

**Table 1:** Overview of Museum-Based Education Programs 2019–2024.


PROGRAM ACADEMIC YEAR	NUMBER OF SESSIONS	SESSION DURATION	TYPE	FORMAT	PARTICIPANTS	TOTAL NUMBER OF PARTICIPANTS	EVALUATION METHOD

2019–2020	6	2 hours	Research Study	In-person	3rd and 4th year medical students	18	Post-session surveys, focus groups

2019–2020	4	2.5 hours	Program	In-person	Pre-health students	23	Post-session surveys

2020–2021 2021–2022 (Course was offered 6 times)	5	2 hours	For-credit course	Online	2nd–4th year medical students	48	Post-session surveys

2021–2022	17	3 hours	For-credit course	Hybrid (12 in-person, 5 online)	Clinical medical students	5	Post-session surveys, reflective essays, interviews

2022–2023 2023–2024	17	3 hours	For-credit course	In-person	Clinical medical students	7 (2022–2023) 5 (2023–2024)	Processfolio, interviews

2023–2024*	13	2.5 hours	For-credit course	In-person	Pre-health upper level students	9	


*No research conducted, only internal program evaluation for the purposes of curriculum refinement.

We aim to share a retrospective synthesis of various iterations of museum-based programs at this institution, as well as specific recommendations for other educators seeking to incorporate museum-based activities into their own initiatives. We also utilize unique findings from more recent museum-based programs that have not previously been published. We have organized our takeaways under the domains of 1) Form, 2) Audience, 3) Function, and 4) Evaluation. Importantly, this paper seeks not only to reaffirm the intrinsic value of museum-based education and review previously described potential benefits and facilitation methods [6,8,9,10,11], but also to address logistic considerations in planning a museum-based program.

## Form

### Size

Considering how the form of museum-based programs can shape the learner experience, we share our experiences implementing different forms of these programs. A group format can facilitate synchronous interactions through promoting “a sense of community… [which] can enhance one’s professional identity and [help] connect with others in [the field]” [6]. This group work aligns with FRAHME recommendations to increase collaboration among scholars and medical professionals [12]. Previous research has found that 8–12 students are ideal for maximizing participation while generating diversity in perspectives [13,14]. In previous JHU offerings, 4–10 students per session have been found to promote robust discussion while still allowing time for everyone’s voice to be included.

### Virtual vs. In-Person

The delivery method of these programs has evolved throughout the pre- and post-pandemic years. Virtual courses can incorporate synchronously experienced multimedia activities (e.g., viewing film clips, listening to music together) and access unlimited works. Moreover, the public nature of museum settings often precludes shared experiences involving audio without relying on individual device accessibility [7]. Virtual offerings may also be more accommodating of learner availability and would not require reimbursement expenses for admission, parking, or transportation.

Notably, students have described how the virtual setting can create a more comfortable environment for sharing insights: “I have a very hard time speaking up in front of others in a group setting and being open with my emotions… but these feelings and discomfort were made acceptable” (previously unpublished feedback from 2021 online course). Another expressed how the online format is “a great way to engage from afar,” and “being at home comes with unique advantages for self-reflection” [7]. Interestingly, virtual course delivery did not detract from students’ sensory experience of the art: “[I] realized how often I don’t pay attention to my senses and live each day taking this for granted”. However, other students have highlighted the power of engaging with the art in-person, describing the physical museum space as “sacred” and distinct from traditional learning places [6].

In post-course surveys of participating students, 64% (n = 7/11) from the first and second offerings of the virtual course [7] and 100% of students (n = 3) from the third offering (previously unpublished feedback from 2020 course), when presented with hypothetical options, preferred a hybrid model to an exclusively virtual or in-person course. However, a subsequent survey of students in a hybrid course (12 sessions in-person, 5 virtual) showed preference for all future sessions to be in-person [9]. We recommend that educators weigh the benefits of pursuing a virtual, hybrid, or fully in-person model, depending on local resources and learners’ experience with in-person courses.

## Audience

While health profession educators should tailor specific course outcomes to different learner stages and expectations [15], museum-based activities can appeal to an array of backgrounds and professional disciplines, as well as patients, families, caregivers, and administrators.

### Learner Background and Motivations

Health professions educators may assume that learners who elect to participate in museum-based activities have arts-affiliated backgrounds. However, motivations are largely mixed. Some indeed have pre-existing familiarity with the arts and humanities through extracurricular or coursework activities. Others approach museum-based programs precisely for their novelty, stating that such coursework “seemed like something different from my norm” and that medical school offered “no other arts/humanities courses.” Educators can augment the perceived value of offerings by emphasizing in program descriptions that no prior arts background or knowledge is required. The museum-based offerings at JHU are elective in nature; mandatory courses may reach a broader audience while potentially limiting the total proportion of engaged learners. Incorporating a brief mandatory museum-based session may pique students’ interests in future elective offerings.

### Learner Stages

It is also important to consider the stage of the learner, whether it be pre-health (at the college level), preclinical or clinical, residency, fellowship, or beyond. Of note, few studies to date have reported on the use of arts and humanities programs at the pre-health and continuing professional development levels [4,8]. In practice, even when museum-based activities were offered as four informal sessions to pre-health students at JHU, participants still reported similar benefits in themes of personal growth, appreciation of multiple perspectives, and cultivation of professional identity as were seen in other formalized offerings [7,11].

It may be especially fruitful to offer these courses during periods of transition, which have been associated with increased feelings of inadequacy, anxiety, or stress for medical students, and can subsequently pose threats to learners’ resiliency and sense of wellbeing [16]. The use of arts and humanities during such periods has been shown to encourage reflection, self-inquiry, and professional identity formation [17]. Educators may consider embedding more museum-based learning activities into program orientations or training sessions in anticipation of transitional stages.

### Clinical Relevance

Maintaining the relevance of clinical connections throughout the program may be most aptly timed during the post-session debrief, when students gather to generate key takeaways, process their emotions, and discuss shared experiences. By intentionally articulating explicit connections to clinical work, trainee roles, or professional trajectories, educators can strengthen the continuity between personal insight and professional transformation, thus fostering increased learner investment in museum-based education [18].

Instructors may also consider incorporating an objective clinical assessment component to improve offerings and demonstrate benefit to learners and patients [3,4]. Although infrequent, some studies report improvements using visual skills examinations of patient photographs [19], radiographic imaging [20] and retinal pathology images [21].

Previous course facilitators and leaders have also developed a digital image library for health profession educators specifically seeking to incorporate visual arts into curricula to address race, racism, and bias [22]; similarly, instructors may intentionally curate pieces with some connection to patient care, though explicit clinical ties are not necessary to generate meaningful discussion.

### Faculty Development

Finally, robust support and training opportunities for faculty are needed to sustain implementation of museum-based programs. This aligns with FRAHME recommendations to provide professional development offerings enhancing faculties’ capacity for curriculum design and use of arts and humanities learning activities [3].

The Harvard Macy Institute’s Art Museum-based Health Professions Education Fellowship demonstrated that immersive faculty development opportunities can deepen interprofessional relationships and create a cohesive community of practice through shared language, frameworks, and approaches to pedagogy and evaluation [23]. Educators must continue to advocate for the recognition of museum-based education by accrediting bodies and funders through allocation of curricular time, resources, and other forms of career advancement, which will ultimately guide mentorship for trainees and legitimize future channels for them to build museum-based education into their professional journey.

## Function

Educators should tailor their program design to the desired function it might serve within the context of learners’ health professions curriculum. The Prism Model suggests that the arts and humanities in medical education can serve four pedagogical functions: skill mastery, perspective-taking, personal insight, and social advocacy [24]. As previously described, focusing on one (or multiple) of these functions can help educators to align their objectives, learning activities and evaluation methods with these selected learning goals [4,24,25]. Here, we will highlight the value of introspection in particular, as well as discussion, in optimizing learner engagement. Reflection can promote wellness and professional identity formation among medical learners [5,17]. In a recent course offering, a majority of students selected “reflection” as their favorite course activity (previously unpublished data). Several students described serving others as part of a “good life,” and that this service was “most achievable if I myself live a good life.” Such reflections align with a core recommendation from the FRAHME report, which encourages integrating arts and humanities-based approaches into medical education to enhance trainee and physician well-being [3].

Learners may benefit from courses with embedded formative intentional reflection, as opposed to a one-time summative reflection at the end. One example of this is through the creation of what we call a processfolio (also known as a process portfolio). A processfolio is a compilation of items such as photographs of objects and creations that document a learner’s personal responses to course activities and assignments. Each item is accompanied by a brief reflection, as well as intermittent formative reflections on various experiences throughout the course. A summative reflection in the form of a self-assessment may also be a component of a processfolio. Both creative and thoughtful, the processfolio reflects a learner’s journey, and may include reflections on what has challenged/confronted/bored them. JHU medical students have appreciated the processfolio, expressing that it “forces [them] to slow down…and think more deeply about not only the art, but also common themes in your life.”

Other reflective components may also be included in museum-based programs, including writing to ambiguous prompts in class and lengthier, graded formative and summative writing assignments outside of class [5,9,11]. Cumulatively, written reflections enable students to explore powerful emotional experiences, reflect on the journey to being a health professional, and weigh the value of personal well-being to themselves and to patients [5]. That being said, we recognize the dangers of methodologically assessing reflection as a “teachable and measurable construct,” which may hinder authentic reflective behavior by reinforcing a “checklist” paradigm [26]. As such, after the first session of each of our for-credit courses at JHU, and as we introduce the written reflective assignments, we assign for homework an article that conveys these ideas to our learners [26]. At the next session, we discuss the article and remind the students that their written reflections will be graded pass/fail on the basis of completion, rather than their ability to conform to a narrow ideal of what “reflection” looks like. When they submit their written reflections, the course director provides feedback that reflects curiosity and conveys appreciation without passing judgment on the reflection’s inherent worth.

## Evaluation

A core FRAHME recommendation for integration of the arts and humanities into medical education is to develop sound methods to evaluate programs and assess learner outcomes [3]. As such, all the JHU museum-based programs described have included formal program evaluations and/or learner assessments. With appropriate Institutional Review Board (IRB) exemption or approval, students can not only sign up for courses for credit but also enroll in them as research participants, thereby maximizing response rates and enabling pre- and post-program comparisons. In this manner, some for-credit programs/courses at JHU were offered as research studies (the JHU IRB reviewed and determined to be exempt all cited studies). Even if not conducting research for publication, understanding learners’ experiences is invaluable for iterative course improvement.

While one-time interventions have been shown to be both feasible and impactful [9], they cannot assess longitudinal benefits or discrete changes in skills and behaviors. In practice, pre-health and medical students at JHU have reported benefits from both one-time and longitudinal offerings [6,8,9,11].

Previous formal program evaluations at JHU have included quantitative and qualitative research via pre- and/or post-surveys that include validated measures as well as open-ended items, focus groups, individual interviews, and analyses of student written reflections [6,7,8]. In all of the qualitative analyses, at least two independent coders compared independently developed codebooks, strengthening the findings.

These experiences suggest that it is possible to design programs in a way that not only includes powerful museum-based learning activities, but also provides opportunities to create text or objects amenable to study as data, such as graded written assignments and processfolios. Recognizing that requiring shared reflections can inhibit their creation, these courses allow students to omit any personal responses from their processfolios before submission.

Educators should also consider incorporating validated psychometric scales that have been used to assess pre-health learners. Commonly utilized scales include the modified Tolerance for Ambiguity scale, the Maslach Burnout Inventory, and the Compassion Scale [9]. Others – such as the Tolerance for Ambiguity scale – are embedded within the AAMC’s Matriculating Student Questionnaire and/or Graduation Questionnaire and therefore enable comparison to broader student populations [27]. A narrative review has explored several other psychometric scales to assess visual arts programming [28].

Although utilizing validated psychometric scales may build a more cohesive body of evidence that promotes generalizability and scalability of museum-based programs, it is also important to account for the inherently versatile landscape of the arts, which merits some flexibility in defining what constitutes “rigor” in evaluation. A purely positivist, metrics-based approach such as Likert scales may privilege a “generalizable simplicity” [29], traditionally associated with the physical sciences, at the expense of valuing the complexity of data drawn from reflection and emotional response. Rather than attempting to leverage evaluation as a way of demonstrating proof, educators should shift towards demonstrating understanding, thus prioritizing process over outcomes.

## Conclusion

Despite an emerging interest in museum-based education, great variability in pedagogy and expertise, as well as insufficient research and evaluation, remain. With an intentionally designed curriculum, the evocative space of the museum can catalyze personal growth and professional development among future health professionals. Possible future directions to be explored in museum-based education include incorporating objective clinical assessments to assess relevance to patient encounters, as well as targeting pre-health students to better understand the value of earlier exposure to museum-based education in their training. This article advocates for wider recognition and integration of museum-based education into the mission and vision of health profession education and provides specific recommendations for implementation across different institutions, offering a spectrum of available resources to support its development.
